# Evaluating Multi-Level Models to Test Occupancy State Responses of Plethodontid Salamanders

**DOI:** 10.1371/journal.pone.0142903

**Published:** 2015-11-30

**Authors:** Andrew J. Kroll, Tiffany S. Garcia, Jay E. Jones, Katie Dugger, Blake Murden, Josh Johnson, Summer Peerman, Ben Brintz, Michael Rochelle

**Affiliations:** 1 Weyerhaeuser, Federal Way, Washington, United States of America; 2 Department of Fisheries and Wildlife, Oregon State University, Corvallis, Oregon, United States of America; 3 U.S. Geological Survey, Oregon Cooperative Fish & Wildlife Research Unit, Department of Fisheries and Wildlife, Oregon State University, Corvallis, Oregon, United States of America; 4 Port Blakely Tree Farms LP, Tumwater, Washington, United States of America; 5 Weyerhaeuser, Lebanon, Oregon, United States of America; 6 Department of Statistics, Oregon State University, Corvallis, Oregon, United States of America; Clemson University, UNITED STATES

## Abstract

Plethodontid salamanders are diverse and widely distributed taxa and play critical roles in ecosystem processes. Due to salamander use of structurally complex habitats, and because only a portion of a population is available for sampling, evaluation of sampling designs and estimators is critical to provide strong inference about Plethodontid ecology and responses to conservation and management activities. We conducted a simulation study to evaluate the effectiveness of multi-scale and hierarchical single-scale occupancy models in the context of a Before-After Control-Impact (BACI) experimental design with multiple levels of sampling. Also, we fit the hierarchical single-scale model to empirical data collected for Oregon slender and Ensatina salamanders across two years on 66 forest stands in the Cascade Range, Oregon, USA. All models were fit within a Bayesian framework. Estimator precision in both models improved with increasing numbers of primary and secondary sampling units, underscoring the potential gains accrued when adding secondary sampling units. Both models showed evidence of estimator bias at low detection probabilities and low sample sizes; this problem was particularly acute for the multi-scale model. Our results suggested that sufficient sample sizes at both the primary and secondary sampling levels could ameliorate this issue. Empirical data indicated Oregon slender salamander occupancy was associated strongly with the amount of coarse woody debris (posterior mean = 0.74; SD = 0.24); Ensatina occupancy was not associated with amount of coarse woody debris (posterior mean = -0.01; SD = 0.29)**.** Our simulation results indicate that either model is suitable for use in an experimental study of Plethodontid salamanders provided that sample sizes are sufficiently large. However, hierarchical single-scale and multi-scale models describe different processes and estimate different parameters. As a result, we recommend careful consideration of study questions and objectives prior to sampling data and fitting models.

## Introduction

Salamanders are a diverse, widespread, and often abundant group of organisms occurring in a broad range of habitat types and serve critical roles in ecosystem dynamics such as nutrient cycling and food webs [1, 2]. Evolutionary studies often use salamanders as subjects because of their novel morphological adaptations, broad geographic distributions, and ancient phylogenies [3–5]. However, life history traits such as limited dispersal and low reproductive rates, and preferences for cool, moist micro-habitats, can render some species vulnerable to habitat modification, exotic species, and disease, and, as a result, many taxa are threatened or imperiled [6–9].

Ecological roles of most salamander species are poorly documented [10, 11]. Due to their multi-stage life histories and occupancy of structurally complex aquatic and terrestrial habitats, studying salamanders poses a unique set of challenges to investigators [12–14]. Long-term studies, preferably with experimental manipulations, are required to understand mechanistic population responses to disturbances events and gradients [11, 15, 16]. However, in most cases only a portion of the population (e.g., in fossorial or in-stream species) is available for sampling [17–20] as many individuals may be sub-surface during sampling. Also, only a portion of these available individuals will be detected due to variation in environmental conditions (e.g., habitat complexity) and animal behavior [21]. Failure to account for this imperfect detection causes bias in naïve estimators of occupancy and abundance [22, 23]. In addition, regression coefficients for associations and/or effects will likely be biased, leading to ineffective or counter-productive conservation and management prescriptions [22]. Finally, disturbances induced by the sampling process can modify salamander habitat and behavior, and these additional sources of variation can bias inferences and future sampling efforts [17]. As a result, sampling methods and estimators of quantities of interest (e.g., occupancy or abundance) require careful thought and evaluation before deployment [24, 25].

We evaluated sampling designs and models for estimating occupancy and detection probabilities for plethodontid salamanders within the context of a planned before-after control-impact manipulative study, and applied them to empirical data on Oregon slender (*Batrachoseps wrighti*) and Ensatina (*Ensatina eschscholtzii*) salamanders as examples. The Oregon slender salamander (BAWR) is endemic to the Oregon Cascades, USA, and is distributed widely in Douglas-fir forests from ~200‒2500 feet in elevation, where the species demonstrates a strong reliance on decayed coarse woody debris [26]. Ensatina salamanders (ENES) are distributed broadly in coniferous and deciduous forests from British Columbia, Canada, to southern California, USA [27].

Occupancy is a state variable of interest in conservation and management studies (e.g., determining species distribution as a function of environmental covariates) and may be defined at different scales. An appropriate choice of scale will depend on factors such as species’ home range and dynamics, scientific or management questions of interest, and sampling constraints [28]. Further, scientific or management interest related to occupancy processes may exist at multiple spatial scales, for example at both local and regional scales [29]. Multi-scale occupancy models are a valuable extension to standard single-scale occupancy models, as they can be used with nested samples to evaluate patterns of habitat selection and use at different spatial scales while accounting for dependencies in occupancy status across scales (i.e., local scale occupancy depends on regional scale occupancy)[30]. Spatio-temporal models are yet another extension of occupancy models that account for variation across time. Several approaches exist to account for temporal variation, including implicit or explicit process dynamics, as well as empirical statistical models used for repeated measures [31–33].

We used simulations to evaluate estimator properties for single-scale and multi-scale occupancy models [29, 34], with empirical correlation structures to account for temporal dependencies, for the analysis of a planned before-after control-impact study. We generated empirical results for the hierarchical single-scale occupancy model using field data for both salamander species and discuss the relative importance of empirical and simulation results in the context of both observational and experimental studies. Researchers and managers can use results from our evaluations to design efficient studies on basic and applied aspects of salamander ecology, conservation, and management. Our results are likely applicable to a broad range of other taxa for which occupancy is evaluated.

## Materials and Methods

### Site Selection & Sampling

We sampled BAWR and ENES at 66 forested harvest units in the Cascade Range, OR, USA. Weyerhaeuser and Port Blakely Tree Farms LP owned the harvest units and provided permission for sampling to occur after reviewing all sampling procedures and experimental manipulations. Field sampling did not involved threatened or endangered species. Harvest unit age ranged from 35–90 ($\bar{x}$ = 60; SD = 8) years and size from 20–183 ha ($\bar{x}$ = 79; SD = 33). Clearcut harvests were used to regenerate all units. Harvest units occurred in one of two study blocks: Clackamas (Clackamas County, OR) or Snow Peak (Linn County, OR)(S1 Fig). We selected harvest units randomly for inclusion within a long-term experimental study of salamander responses to contemporary management prescriptions.

Within each harvest unit, we sampled seven 81 m^2^ (9×9 m) plots in 2013–2014. We chose plot sizes to incorporate multiple salamander home ranges [28] and to allow systematic searching of habitat features. We selected each 81 m^2^ plot randomly and sampled over three consecutive 10 minute intervals in a single day (sampling occurred between 0800–1600 and from April-June in 2013 and 2014). During each 10 minute interval, one observer surveyed the plot. Although the same harvest units were sampled in both years, we selected a new set of plots to sample in each year (i.e., each plot received a maximum of three 10 minutes surveys). Spatial and temporal replication was necessary to estimate and incorporate detection into estimates of occupancy [35]. Observers employed a light touch methodology [36, 37] in which all surface objects, including leaf litter and moss blankets on logs, were turned over to observe salamanders. All surface objects were then returned to as close to their original position as possible so as to not negatively impact the habitat quality within a plot for salamanders. Observers did not survey the same objects during each interval, but aimed to sample the entire plot over the course of the three intervals. Salamanders were not handled with this survey method. We followed a removal sampling protocol in which sampling stopped once both species were observed in a plot [31]. During sampling, observers quantified number of pieces of coarse woody debris (CWD; all logs > 25 cm DBH (small end) and > 1 m in length).

### Statistical Models

The sampling design described above incorporates replication at two spatial scales (unit-level and plots within units) and two temporal scales (year and visit). Our objective in formulating a statistical model was to estimate patterns of occupancy at relevant scales of interest while accounting for the sampling design and adjusting for imperfect detection. To this end, we considered two different statistical models each representing a distinct occupancy process.

The first approach, which we refer to as the “hierarchical single-scale model”, is a modified version of the basic MacKenzie occupancy model [35], that incorporates random effects to account for both the nested spatial sampling design and repeated measurements from the same unit across time. Specifically, we let *u*_*ijk*_ denote the occupancy of plot *k* in year *j* from harvest unit *i*, which takes the value of 1 when the plot is occupied and 0 otherwise. We assume that plot-level occupancy follows a Bernoulli distribution *u*_*ijk*_ ∼ *Bern*(*ψ*_*ijk*_), where *ψ*_*ijk*_ is the probability that plot *k* of harvest unit *i* during year *j* is occupied. Within-plot occupancy is assumed to be closed across all visits within a year. We further assume that occupancy probability is defined in terms of a mean model, *μ*_*ijk*_, and a unit-specific random intercept, *b*_0*i*_: *logit*(*ψ*_*ijk*_) = *μ*_*ijk*_ + *b*_0*i*_. The mean model may be parameterized in terms of harvest unit level, year level and plot level covariates, while the random effect imparts a correlation among plots within a harvest unit and among measurements on the same harvest unit over time. We assume independence among plots in different harvest units, and conditional independence among plots within the same harvest unit, given the unit-level random effects. For the detection process, we let *y*_*ijkl*_ denote the detection status of plot *k* from harvest unit *i* during year *j* and visit *l*, taking the value of 1 when the species is detected and 0 otherwise. We considered species detection to also follow a Bernoulli distribution *y*_*ijkl*_ = *Bern*(*u*_*ijk*_ ⋅ *p*_*ijkl*_), with detection probability *p*_*ijkl*_.

The second approach, which we refer to as the “multi-scale” model, is a modified version of methods for multi-scale inference introduced in [29, 34]. In this approach, we allow for two nested but distinct occupancy processes, one acting at the harvest unit level, and the other acting at the plot level, conditional on harvest unit occupancy. We let *z*_*ij*_ denote the occupancy of harvest unit *i* in year *j*, which takes the value of 1 when the harvest unit is occupied and 0 otherwise. We assume that *z*_*ij*_ follows a Bernoulli distribution *z*_*ij*_ ∼ *Bern*(*θ*_*ij*_) with harvest unit occupancy probability *θ*_*ij*_. Similar to plot-level occupancy for the single-scale model, we parameterize unit-level occupancy probability in terms of a mean model *μ*_*ij*_ and a unit-specific random intercept, *b*_0*i*_: *logit*(*θ*_*ij*_) = *μ*_*ij*_ + *b*_0*i*_. The random intercept in this model accounts for repeated measurements on the same harvest unit across years. We represent the plot-level occupancy as a Bernoulli random variable *u*_*ijk*_ ∼ *Bern*(*z*_*ij*_ ⋅ *ψ*_*ijk*_). Under this parameterization, plot-level occupancy probability is equal to *ψ*_*ijk*_ when *z*_*ij*_ = 1, and equal to 0 when *z*_*ij*_ = 0. The detection process is again considered to follow a conditional Bernoulli distribution *y*_*ijkl*_ = *Bern*(*u*_*ijk*_ ⋅ *p*_*ijkl*_), with detection probability *p*_*ijkl*_, and where *y*_*ijkl*_ = 1 if the species is detected and 0 otherwise. Covariates may be included in any of these models, but are restricted to harvest unit and year variables for *θ*_*ij*_, to plot, year or harvest unit variables for *ψ*_*ijk*_, and visit, plot, year, or harvest unit variables for *p*_*ijkl*_. Under this model, we assume independence among harvest units and conditional independence on measurements of the same harvest unit over time, given the unit-specific random effects. Plots within a harvest unit are also considered conditionally independent given the partially latent occupancy status *z*_*ij*_.

Both the hierarchical single-scale and multi-scale models may contain effects acting at different scales. The primary distinction between the two models is the scale at which an assumed occupancy process manifests: strictly at the plot level for the hierarchical single-scale model or separately at both the plot and harvest unit levels for the multi-scale model. For example, in the hierarchical single-scale model, we assume that habitat selection is taking place at the plot scale only, but that factors at a larger scale (the harvest unit) may influence this selection. For the multi-scale model, we assume that two different selection decisions are taking place: at the harvest unit first, and conditional on a harvest unit being selected, a second selection decision is made at the plot scale. Given this distinction, we cannot directly contrast the performance of these models. Instead, we present the results here for each model as two distinct options to be considered for analysis.

### Simulation Study

We conducted a simulation study to evaluate the effectiveness of each occupancy model in the context of a Before-After Control-Impact (BACI) [38] experimental design with multiple levels of sampling. Our goal was to assess the impact of sample size on the consistency and efficiency of the treatment effect estimator of a BACI analysis, and to aid in the design of a planned manipulative experiment. We simulated data for both the hierarchical single-scale process and the multi-scale process discussed in the previous section. Models were then fit to data simulated under the assumed model (e.g., the multi-scale model was only fit to data simulated from the multi-scale process). The impact of model misspecification and checks for model misspecification were not considered in this study.

To simulate a BACI design, we assumed that *N* harvest units would be split evenly among two treatments (“control” and “impact”), and would be followed for two years: one year “before” and one year “after”. Furthermore, *n* plots were assumed to be placed within each of the *N* harvest units, with each plot visited *m* times. In this setting, harvest units represent our primary sampling units and are the experimental units with respect to the treatment. Plots within harvest units are a secondary sampling unit and represent sub-samples with respect to the applied treatment. As with our empirical study, we assumed that different plots within a harvest unit were sampled across years and could therefore be considered conditionally independent sampling units. Unlike our empirical study, the simulation study did not stop visits after the first detection.

The mean model used to simulate occupancy data from the hierarchical single-scale model was of the form:

$$logit\left(\psi_{ij}\right)=\beta_{0}+b_{0i}+\beta_{1}{\text{Year}}_{ij}+\beta_{2}{\text{Treatment}}_{i}+\beta_{3}{\text{Treatment}}_{i}\cdot {\text{Year}}_{ij},$$ (1)

where *ψ*_*ij*_ is the expected plot-level occupancy of year *j* in harvest unit *i*, *Year*_*ij*_ is an indicator variable for Year = “after”, and *Treatment*_*i*_ is an indicator variable for “impact” stands. The random intercept *b*_0*i*_ is assumed to follow a normal distribution with mean = 0 and variance = $\sigma_{b}^{2},$ and is included to account for correlation among plots within a harvest unit. Under this occupancy parameterization, *exp*(*β*_3_) is interpreted as the relative effect of the impact treatment on plot-level occupancy-odds, after controlling for year and stand-level variation. Thus, the parameter *β*_3_ can be considered an estimator for the impact treatment effect. For simplicity, no covariates, including treatment effects, were included in the detection probability model.

The mean model used to simulate data from the multi-scale model was of the form:

$$logit\left(\theta_{ij}\right)=\alpha_{0}+\alpha_{0,i}+\alpha_{1}{\text{Year}}_{ij}+\alpha_{2}{\text{Treatment}}_{i}+\alpha_{3}{\text{Treatment}}_{i}\cdot {\text{Year}}_{ij},$$ (2)

where *θ*_*ij*_ is the expected harvest unit-level occupancy of harvest unit *i* in year *j*, Year_*ij*_ is an indicator variable for Year = “after”, and Treatment_*i*_ is an indicator variable for “impact” harvest units. We used constant values for both the conditional plot-level occupancy, *ψ*_*ijk*_, and detection probability, *p*_*ijkl*_, for plot *k* and visit *l*. As with the hierarchical single-scale model, the random intercept *a*_0,*i*_ is assumed to follow a normal distribution with mean = 0 and variance = $\sigma_{b}^{2}.$ Under this parameterization, *exp*(*α*_3_) is interpreted as the relative effect of the impact treatment on harvest unit-level occupancy-odds, after controlling for year and harvest unit-level variation.

We draw attention to the different interpretations of the “treatment effect” in each of these models. Under the hierarchical single-scale model, the treatment effect is estimated for plot level occupancy, whereas under the multi-scale model, the effect is estimated for harvest unit-level occupancy. The two occupancy models correspond to distinct occupancy processes with different interpretations. Consequently, we used different parameter values in our simulation study for the two different models. In each case, we chose parameters to fall within an expected range for our application to BAWR and ENES (Table 1).

**Table 1:** Parameters used to simulate data under the hierarchical single-scale and multi-scale occupancy models.

Parameter description	Hierarchical single-scale model	Multi-scale model
Number of stands	20, 30, 40, 50, 60	20, 30, 40, 50, 60
Number of plots/stand	5, 7, 9	5, 7, 9
Number of visits/plot	3	3
Pre-treatment stand occupancy	NA	0.95
Pre-treatment plot occupancy	0.7	0.5
Post-treatment stand occupancy	NA	0.3, 0.6
Post-treatment plot occupancy	0.1, 0.3	0.5
Detection probability	0.15, 0.3, 0.5	0.15, 0.3, 0.5

We evaluated all parameter combinations in Table 1, giving a total of 90 unique conditions for each model. Each unique condition was simulated 500 times. All datasets were simulated in R [39] and we fit all models using JAGS [40] called from R using package ‘R2jags’ [41]. For both models, we used 3 chains of length 10000 each, with a burnin of 5000 and 1/10 thinning. We checked convergence for occupancy and detection model parameters using the Gelman-Rubin statistic [42]. The results summary included only those simulations where all mean model convergence statistics were less than 1.1. We included the R code used to conduct the simulation study in S1 and S2 Files.

### Empirical Analysis

We chose to fit the hierarchical single-scale model to the empirical data collected across two years on 66 harvest units in the Cascade Range, Oregon, USA. These data represent pre-treatment conditions in harvest units that will be included in a long-term BACI design to investigate the impact of timber harvest on BAWR and ENES occupancy. Our goals in this analysis were to obtain preliminary estimates of salamander plot-level occupancy in control conditions; to understand the association between occupancy and coarse woody debris; and to estimate detection probabilities. We could have chosen to fit the multi-scale model to our dataset. However, we selected randomly harvest units to be included in the study from a larger pool of harvest units, all of which were known to be occupied. Consequently, no variation existed in occupancy status at the harvest unit level, and we could not model the upper-level occupancy process. For both BAWR and ENES, we modeled plot-level occupancy as a function of CWD, Year, and Block:

$$logit\left(\psi_{ij}\right)=\beta_{0}+b_{0i}+\beta_{1}{\text{CWD}}_{ij}+\beta_{2}{\text{Year}}_{ij}+\beta_{3}{\text{Block}}_{i},$$ (3)

where CWD_*ij*_ is the coarse woody debris count for plot *j* in unit *i*, Year_*ij*_ is an indicator for whether plot *j* was sampled in 2014, and Block_*i*_ is an indicator for harvest units in the Clackamas tree farm. We sampled coarse woody debris on plots each year during salamander sampling.

Detection probability was modeled as a quadratic function of Julian date and an indicator variable for year:

$$logit\left(p_{ijk}\right)=\gamma_{0}+\gamma_{1}{\text{Julian Date}}_{ijk}+\gamma_{2}{\text{Julian Date}}_{ijk}^{2}+\gamma_{3}{\text{Year}}_{ij},$$ (4)

where Julian Date_*ijk*_ is the Julian date for visit *k* of plot *j* in harvest unit *i*, and Year_*ij*_ is an indicator for whether plot *j* was sampled in 2014. We centered and scaled Julian date prior to analysis.

We fit all models in a Bayesian framework using *N*(*μ* = 0, *σ* ^2^ = 3) priors for the plot-level occupancy and detection probability intercepts, and *N*(*μ* = 0, *σ* ^2^ = 4) priors for occupancy and detection covariates. A *Gamma*(2,1) prior was used for the random effects standard deviation. We fit the models using JAGS, called from R, with three chains of length 55,000, a burnin of length 5,000, and 1/10 thinning. We assessed convergence using the Gelman-Rubin statistic and visual inspection of the chains [42]. The JAGS model used for this analysis is included in S3 File. The detection data and covariates for both BAWR and ENES are included in S1 and S2 Tables.

### Ethics Statement

We conducted this research in compliance with all Oregon and USA laws and regulations. The Oregon State University Institutional Animal Care and Use Committee (IACUC) approved all activities involving the sampling and handling of live vertebrate animals.

## Results

### Simulations

We used the results of the simulation study to focus on three quantities of interest related to the treatment effect estimator: bias, precision, and coverage probability. We calculated bias as the difference between the true value of the parameter and the expected value of the estimator, and (inverse) precision as the mean posterior standard deviation of the treatment estimator. Coverage probability was calculated as the proportion of times a 95% equal-tail credible interval contained the true value of the treatment effect parameter. Bayesian posterior credible intervals often show close to nominal coverage probabilities in both small and large sample problems [42, 43], and we examine their performance in our results.

Although we fit our models in a Bayesian framework, we would like to understand these frequentist properties of the model estimators, as they provide useful pre-experimental guidance on the use of the models and design considerations. Any Bayesian estimate will, to some extent, depend on the priors used in the analysis. Nevertheless, we consider it instructive to examine these pre-experimental properties of the model estimators, even under a limited set of conditions.

**Fig 1:**
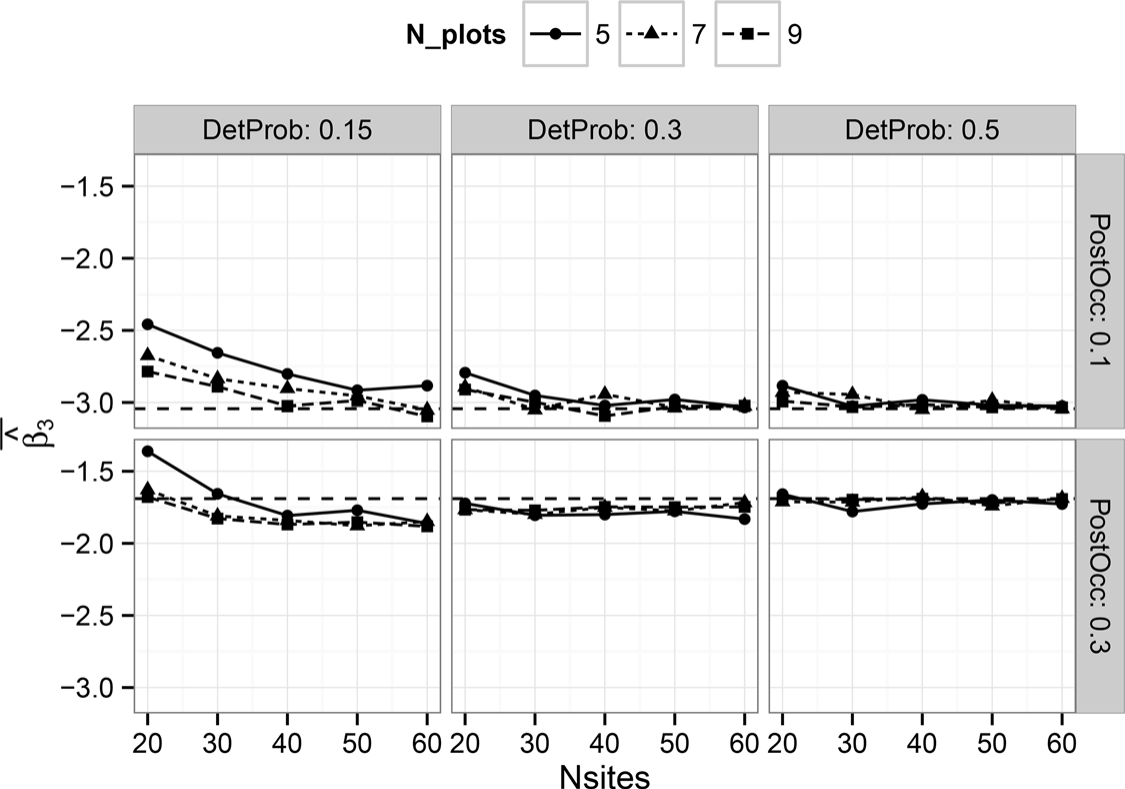
Average, by combination of parameters, of all posterior mean estimates of the treatment effect estimator ‘Beta3’ (see Eq 1) in the hierarchical single-scale occupancy model. Results are shown on the logit scale. Panels show the results for different combinations by simulated detection probability and post-treatment occupancy. Horizontal dashed lines show the true coefficient value.

**Fig 2:**
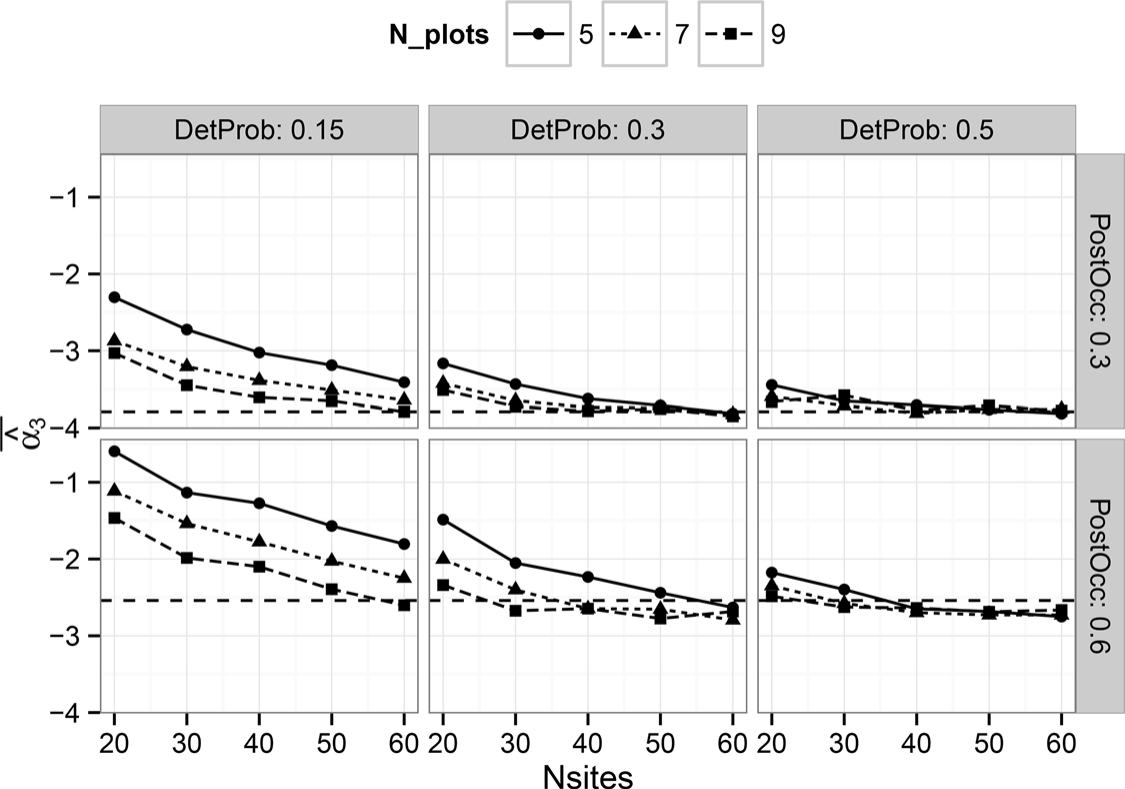
Average, by combination of parameters, of all posterior mean estimates of the treatment effect estimator ‘Alpha3’ (see Eq 2) in the multi-scale occupancy model. Results are shown on the logit scale. Panels show the results for different combinations by simulated detection probability and post-treatment occupancy. Horizontal dashed lines show the true coefficient value.

The hierarchical single-scale model showed consistent results for the average posterior mean of the treatment effect estimator for all sample sizes when detection probability was 0.5, but some evidence of bias when detection probability was 0.15 (Fig 1). Bias was generally small (<10%) for all detection probabilities when the number of harvest units was at least 40. Estimator bias for the multi-scale model was strongly dependent on both the number of simulated harvest units as well as the number of plots per harvest unit, particularly at lower detection probabilities (Fig 2). Although bias was generally low when detection probability was 0.5, substantial bias existed with low detection probabilities, particularly in cases with less than 50 harvest units (Fig 2). However, these trends suggest that with sufficient sample size at both the primary and secondary sampling units, approximately unbiased estimates can be obtained for the treatment effect estimator under either model in a BACI design, at least for the prior distributions and range of parameter values considered in this study.

**Fig 3:**
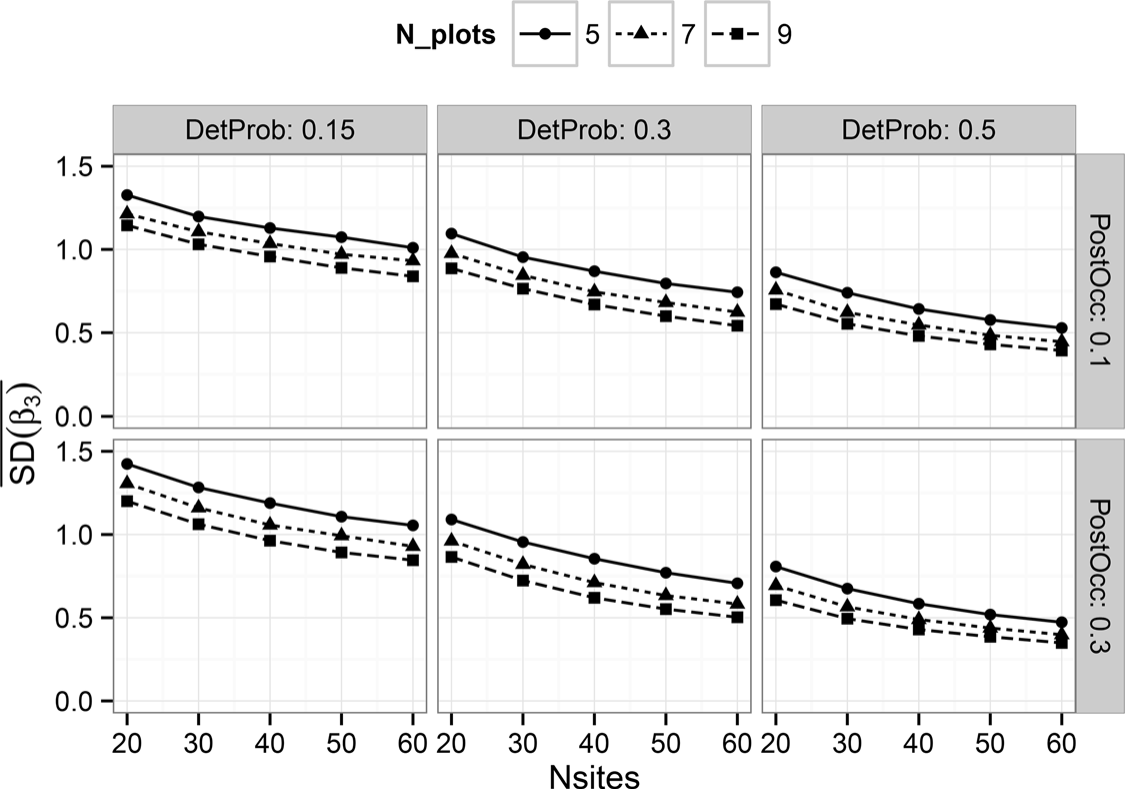
Average standard deviation, by combination, of all posterior mean estimates of the treatment effect estimator ‘Beta3’ (see Eq 1) in the hierarchical single-scale model. Results are shown on the logit scale. Panels show the results for different combinations by simulated detection probability and post-treatment occupancy.

**Fig 4:**
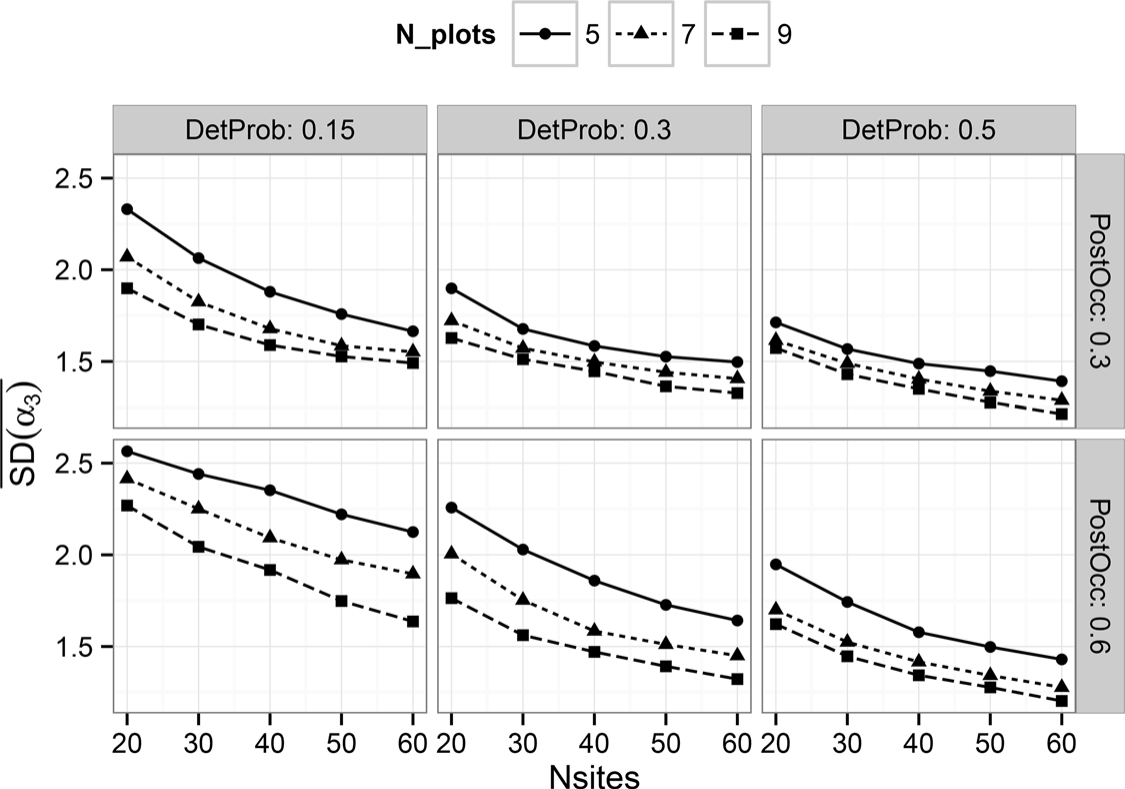
Average standard deviation, by combination, of all posterior mean estimates of the treatment effect estimator ‘Alpha3’ (see Eq 2) in the multi-scale model. Results are shown on the logit scale. Panels show the results for different combinations by simulated detection probability and post-treatment occupancy.

We observed improved posterior estimator precision with increasing numbers of harvest units, as expected (Figs 3 and 4). Number of plots per harvest unit, which represent subsamples, also played a role in estimator precision by affecting the amount of information available to estimate occupancy of the primary sampling unit. These trends also suggest improved precision with increasing detection probability, but very little impact due to the size of the treatment effect.

**Fig 5:**
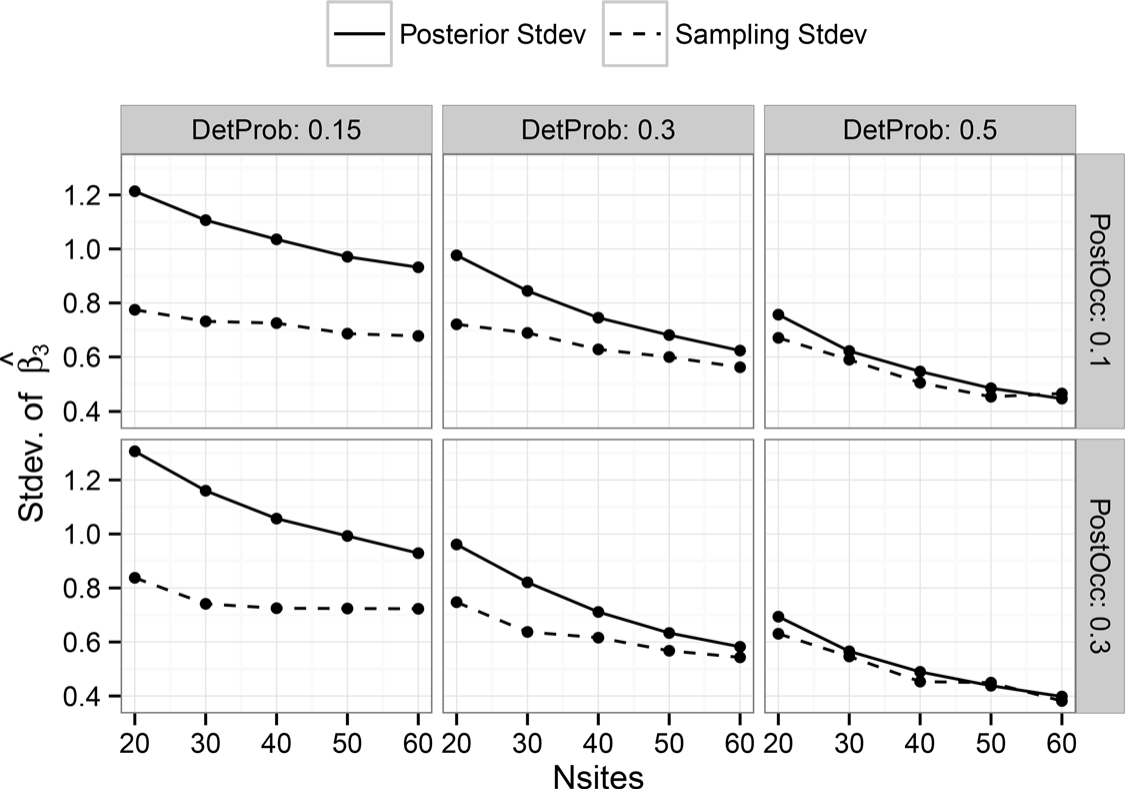
Comparison of the standard deviation of posterior mean estimates of ‘Beta3’ vs. the average posterior standard deviation of ‘Beta3’ for the hierarchical single-scale model. Results are shown only for combinations with seven plots. Results for five and nine plots are not shown, but have trends similar to those shown here. Panels show the results for different combinations of detection probability and post-treatment occupancy.

**Fig 6:**
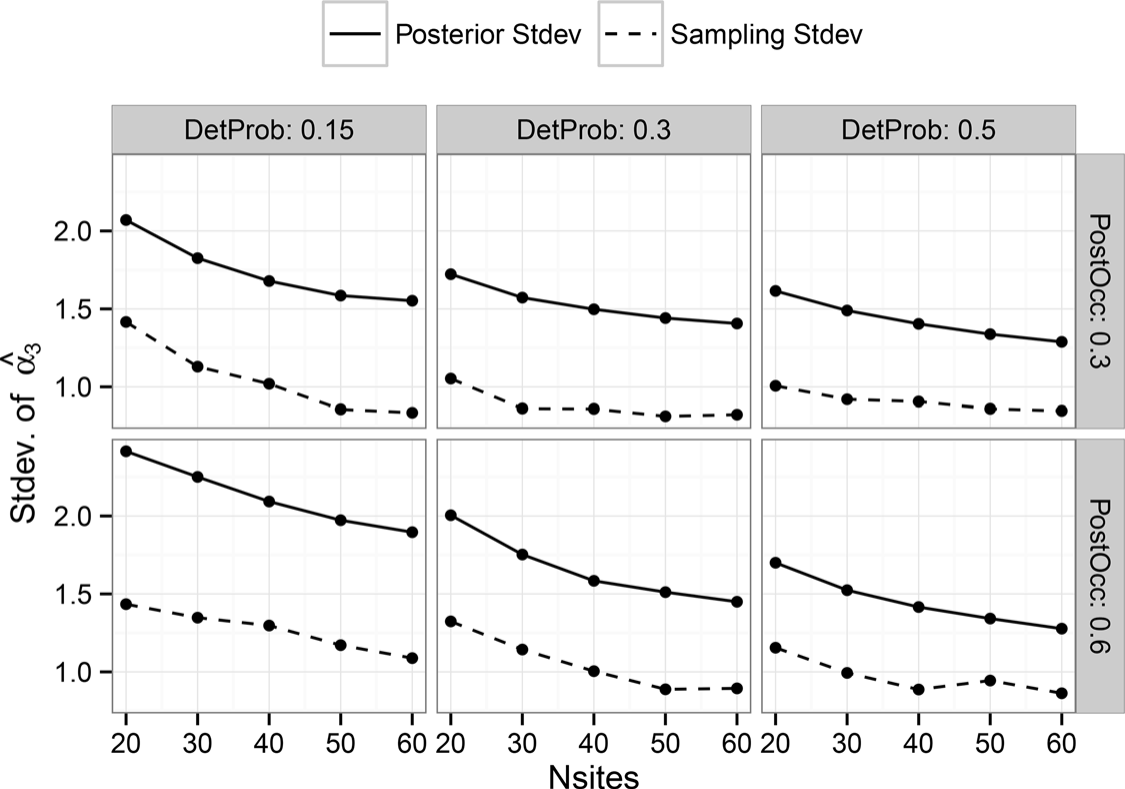
Comparison of the standard deviation of posterior mean estimates of ‘Alpha3’ vs. the average posterior standard deviation of ‘Alpha3’ in the multi-scale model. Results are shown only for combinations with seven plots. Results for five and nine plots are not shown, but have trends similar to those shown here. Panels show the results for different combinations of detection probability and post-treatment occupancy.

Coverage probabilities for the single-scale model treatment effect estimator (Table 2, S3 Table) were close to nominal, but somewhat conservative with an average value of 0.97. Coverage probabilities for the multi-scale model treatment effect estimator were highly conservative, with 100% of all intervals containing the true value. This result indicates that the posterior standard deviation of the treatment effect parameter is not a good surrogate for the standard deviation of the estimator sampling distribution. The hierarchical single-scale model (Fig 5, S3 Table) showed generally good calibration between sampling variability and posterior estimates of variability when detection probability was 0.5 for all sample sizes considered in this study. As detection probability decreased, posterior variability tended to be greater than the sampling variability of the treatment estimator. Results from the multi-scale model show, across all conditions included in this study, that the average posterior variability is substantially greater than the sampling variability of the treatment estimator (Fig 6). The trends for the multi-scale model (S4 Table) suggest that posterior credible intervals for the treatment effect estimate will tend to be highly conservative, exceeding nominal coverage rates and limiting the efficiency of estimating the sign and magnitude of putative treatment effects.

**Table 2:** Estimated coverage probabilities for nominal 95% equal-tail credible intervals of the treatment effect estimator from hierarchical single-scale and multi-scale occupancy models. Results in this table are limited to combinations with detection probability of 0.3 and seven plots. Results for other conditions are consistent with these trends (S3 and S4 Tables).

Number of plots	Single-scale model coveragePost-treatment occupancy = 0.1	Single-scale model coveragePost-treatment occupancy = 0.3	Multi-scale model coveragePost-treatment occupancy = 0.3	Multi-scale model coveragePost-treatment occupancy = 0.6
20	0.97	0.98	1.00	1.00
30	0.98	0.99	1.00	1.00
40	0.96	0.98	1.00	1.00
50	0.96	0.96	1.00	1.00
60	0.97	0.97	1.00	1.00

**Fig 7:**
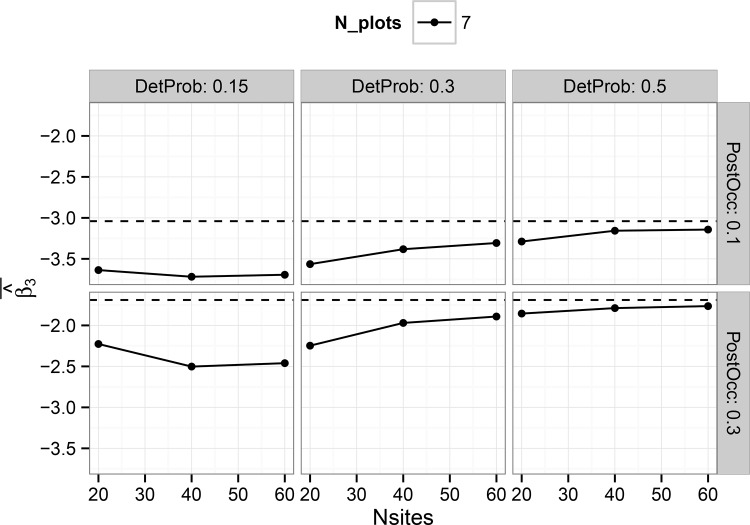
Average, by combination of parameters, of all posterior mean estimates of the treatment effect estimator ‘Beta3’ (see Eq 1) in the hierarchical single-scale occupancy model under a relaxed set of priors. Note we ran only a subset of cases in Table 1 for this analysis. Results are shown on the logit scale. Panels show the results for different combinations by simulated detection probability and post-treatment occupancy. Horizontal dashed lines show the true coefficient value. Prior sensitivity can be assessed by comparing with Fig 1.

**Fig 8:**
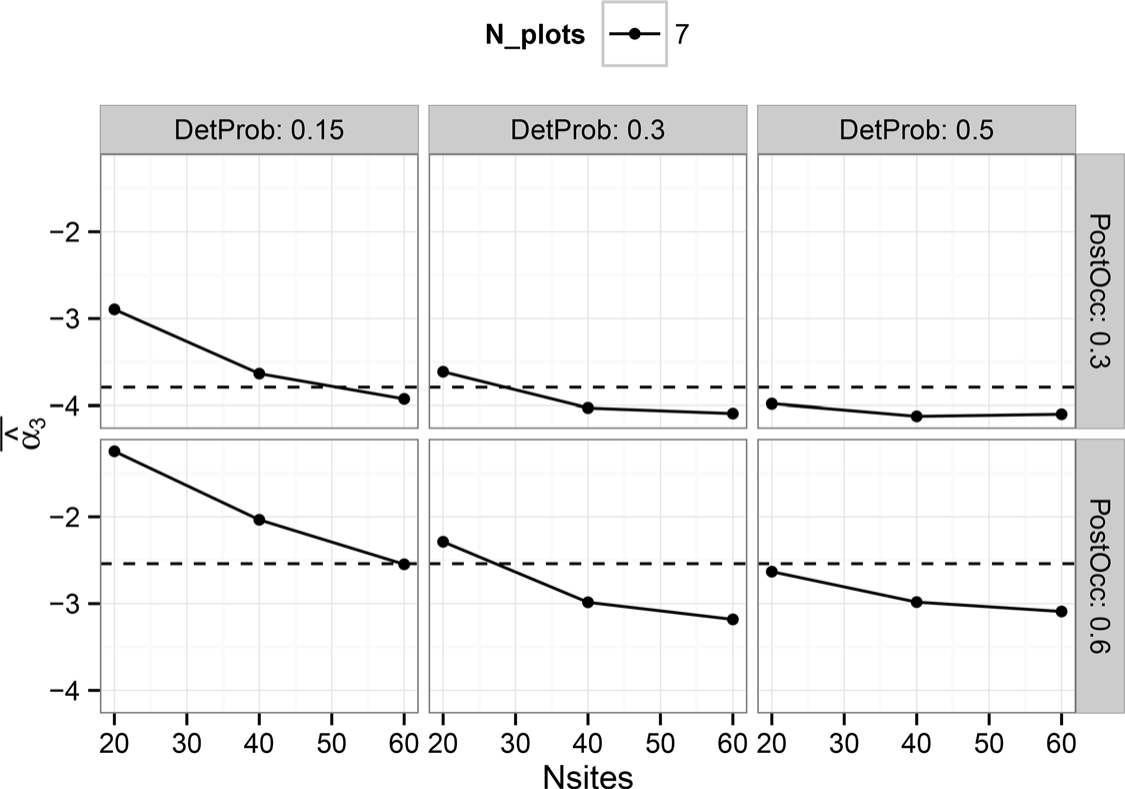
Average, by combination of parameters, of all posterior mean estimates of the treatment effect estimator ‘Alpha3’ (see Eq 2) in the multi-scale occupancy model under a relaxed set of priors. Note we ran only a subset of cases in Table 1 for this analysis. Results are shown on the logit scale. Panels show the results for different combinations by simulated detection probability and post-treatment occupancy. Horizontal dashed lines show the true coefficient value. Prior sensitivity can be assessed by comparing with Fig 2.

All of the above results are based on a single set of prior distributions, chosen beforehand to reflect subject matter knowledge regarding plethodontid salamander behavior. Although we think that this choice of priors is appropriate for our current application, these priors are potentially informative and have the potential to affect estimator properties such as bias and precision. At the suggestion of a reviewer, we repeated our simulation study with relaxed priors—*N*(*μ* = 0, *σ* ^2^ = 10) for covariate parameters—for a reduced set of the conditions in Table 1. Results for this simulation study are summarized in S5 and S6 Tables. The hierarchical single-scale model with relaxed priors showed a negative bias in the treatment estimator expected value across most conditions (Fig 7). The magnitude of bias was affected by both sample size and detection probability, showing relatively small bias with moderate detection probabilities and larger sample sizes, and substantial bias with low detection probabilities. Results for the multi-scale model with relaxed priors showed a mix of positive and negative bias depending on the simulation conditions (Fig 8). Under both the single-scale and multi-scale models, estimator precision and coverage probabilities using the relaxed priors were substantially the same as with our original choice of priors (S5 and S6 Tables).

### Empirical Evaluations

Estimated plot-level mean occupancies from the hierarchical single-scale model were higher in the Clackamas tree farm than the Snow Peak tree farm for BAWR (0.66 vs. 0.43), but lower for ENES (0.20 vs. 0.63)(Table 3). The results also showed a strong association between coarse woody debris count and BAWR plot-level occupancy. We estimated approximately 35% higher odds of occupancy for each additional piece of CWD found in a plot. In contrast, no clear association existed between ENES occupancy and CWD.

**Table 3:** Posterior estimates of model parameters and standard deviations (SD) for both Oregon slender (BAWR) and ensatina (ENES) salamanders, Oregon Cascades, USA, 2013–2014. All estimates are reported on the logit scale to two significant digits. Estimates for coarse woody debris (CWD) and Julian date (JD) are based on standardized covariates.

		BAWR		ENES	
Model	Term	Mean	SD	Mean	SD
Occupancy	Intercept	-0.26	0.48	0.52	0.78
	Block = Clackamas	0.93	0.41	-1.9	0.72
	Year = 2014	0.012	0.55	2.1	1.1
	CWD	0.74	0.24	-0.01	0.29
Detection	Intercept	-0.69	0.38	-1.8	0.33
	JD	0.22	0.11	-0.20	0.10
	JD^2^	-0.031	0.11	0.035	0.11
	Year = 2014	-0.64	0.38	-0.58	0.34

Detection probabilities were estimated to be lower in 2014 than in 2013 for both BAWR (0.21 vs. 0.33) and ENES (0.09 vs. 0.14)(Table 3). Detection probabilities tended to increase later in the season for BAWR, but decrease later in the season for ENES.

## Discussion

Diverse life history strategies and behavioral variation make Plethodontid salamanders challenging organisms to study [17, 19, 44]. Evaluation of sampling designs and estimators for responses of interest are critical to provide strong inference about Plethodontid ecology and responses to conservation and management activities. We used simulations to evaluate performance of two candidate occupancy models in an analysis of a BACI experiment, and to set appropriate sample sizes for the experiment. Our results indicated expected improvements in precision with increasing numbers of primary sampling units, but also highlighted the potential gains accrued when adding secondary sampling units. Importantly, our results identified conditions that could suffer from excessive estimator bias under particular choices of prior distributions. Both models showed evidence of estimator bias at low detection probabilities and low sample sizes, a problem that was also dependent on the choice of prior distribution. However, the results also suggested that sufficient sample size at both the primary and secondary sampling levels could ameliorate this issue.

Another insight from this study relates to the calibration between posterior uncertainty and sampling variability. Under some conditions, good calibration existed between these two quantities for the hierarchical single-scale model, suggesting that posterior credible intervals could have a similar pre-experimental interpretation as confidence intervals. However, in all cases studied for the multi-scale model and in many cases for the hierarchical single-scale model, the posterior variability was substantially greater than the sampling variability. Under these conditions, interpreting credible intervals as confidence intervals appears unjustified and Bayesian interpretation of results is preferred.

Based on the simulation results, we expanded the size of our multi-year BACI experiment from 45 to 66 harvest units and maintained seven plots per harvest unit. This change should help to reduce estimator bias and improve precision.

The results of the simulation study are limited in several ways. Our study did not fully address the sensitivity of results to the choice of priors. Our limited comparison of two different prior distributions highlights sensitivity of estimator properties to these choices, even with priors that may be considered ‘reasonable’ based on subject matter considerations. We therefore stress the importance of evaluating model performance within applied contexts prior to fitting the model to data, as well as the need for posterior model checking.

We did not consider issues of lack-of-fit, variable selection, or the impact of model misspecification on estimator performance. These issues are important, but we considered them to be outside the scope of the study. In our particular application, some of these issues are less of a concern, as certain aspects of the model will be determined by the sampling design. For example, all design-based factors will be included in the occupancy model(s), using a pre-determined functional form, regardless of statistical ‘significance’.

We did not address the question of how one might select between the two models that we explored in the simulation study. As the two models represent two distinct processes for occupancy, scientific judgment can and should be considered in the choice of model. In cases such as our empirical example, the choice of models may be guided by species distribution considerations apparent in the raw data. When limited background information is available to support one model over the other, or when a goal of the analysis is to infer which model is a better descriptor of the data, a range of options are available for Bayesian model selection and comparison [45, 46]. In all cases, it is incumbent on the analyst to perform appropriate diagnostic checks.

In the empirical analysis, we wanted to obtain preliminary estimates of salamander occupancy and detection under control conditions; examine the association between salamander occupancy and coarse woody debris; and determine suitability of models for evaluation of data collected within a BACI experimental design. Our results suggest higher plot-level occupancy of Oregon slender salamander than ENES at Clackamas; the reverse is true for Snow Peak. Our results also helped to quantify the positive association between BAWR occupancy and CWD count, a trend which had been observed during stand pre-selection. Oregon slender salamanders are thought to rely on CWD for foraging and nesting substrates, as opposed to the more generalist affinities of ENES [26]. However, we note that both BAWR and ENES appear to be relatively abundant on our study sites. For organisms occurring as relatively abundant and well-distributed populations, estimating effects of habitat change may be difficult if the response variable is occupancy as sample units may remain occupied even if population size changes substantially.

Finally, the detection probability estimates for both BAWR and ENES were similar to conditions examined in the simulation study, and suggest low bias for the BACI treatment-effect estimator under either model (and our choice of priors), given that we have included 66 harvest units in our study. However, due to relatively low detection probabilities, we can anticipate that the multi-scale model treatment-effect credible intervals for ENES will be substantially larger than the estimator sampling variability. Consequently, the intervals can be expected to exceed nominal coverage, and will be conservative if used to assess evidence of a putative treatment effect.

## Supporting Information

Additional supporting information may be found in the online version of this article.

S1 Fig Geographic distribution of Oregon slender (*Batrachoseps wrighti*) and Ensatina (*Escholtzii ensatina*) salamanders and ownership boundaries within study area, Oregon, USA, 2013–2014. The Clackamas block occurs in Clackamas County; the Snow Peak block occurs in Linn County.
(EPS)

S1 File R code for the simulation study for 'hierarchical single-scale’ BACI occupancy model. (DOCX)

S2 File R code for the simulation study for 'multi-scale' BACI occupancy model. (DOCX)

S3 File R code for empirical analyses of Oregon slender and ensatina salamander occupancy, Oregon Cascades, US, 2013–2014. (DOCX)

S1 Table Detection data and covariates for Oregon slender salamanders, Oregon Cascades, USA, 2013–2014. (CSV)

S2 Table Detection data and covariates for Ensatina salamanders, Oregon Cascades, USA, 2013–2014. (CSV)

S3 Table Full results of simulation study for ‘hierarchical single-scale’ BACI occupancy model. (CSV)

S4 Table Full results of simulation study for ‘multi-scale’ BACI occupancy model. (CSV)

S5 Table ‘Hierarchical’ BACI occupancy model simulation summary with alternative priors. (CSV)

S6 Table ‘Multi-scale’ BACI occupancy model simulation summary with alternative priors. (CSV)
