# Correction: Evaluating Multi-Level Models to Test Occupancy State Responses of Plethodontid Salamanders

**DOI:** 10.1371/journal.pone.0145899

**Published:** 2015-12-23

**Authors:** The PLOS ONE Staff

There is an error in the seventh author’s name. The correct name is: Summer Peterman. The publisher apologizes for the error.

## References

[1] Kroll AJ, Garcia TS, Jones JE, Dugger K, Murden B, Johnson J, et al (2015) Evaluating Multi-Level Models to Test Occupancy State Responses of Plethodontid Salamanders. PLoS ONE 10(11): e0142903 doi:10.1371/journal.pone.0142903 26619010 10.1371/journal.pone.0142903 PMC4664280

