# Supplementary material for: Evaluating Multi-Level Models to Test Occupancy State Responses of Plethodontid Salamanders
Source: PLoS One. 2015 Nov 30;10(11):e0142903. doi: 10.1371/journal.pone.0142903 (PMC4664280; doi:10.1371/journal.pone.0142903)
Supplement: S1 File — (DOCX) [file pone.0142903.s002.docx]

**S2 File:** R code for the simulation study for 'hierarchical’ BACI occupancy model.

#

# Simulation study for 'hierarchical' BACI occupancy model

#

library(R2jags)

library(plyr)

# ------------------------------

# 1. JAGS model

# ------------------------------

# hierarchical occupancy model

model.h <- function(){

# priors

aInt.mean ~ dnorm(0,0.25)

aInt.sd ~ dgamma(2, 1)

aInt.tau <- 1/(aInt.sd * aInt.sd)

bTrt ~ dnorm(0, 0.25)

bYr ~ dnorm(0, 0.25)

bTrtYr ~ dnorm(0, 0.25)

gInt ~ dnorm(0, 0.333)

# stand-level model

for(i in 1:R){

b0[i] ~ dnorm(aInt.mean, aInt.tau)

}

for(i in 1:N){

bMean[i] <- b0[StandID[i]] + bTrt*Trt[i] + bYr*Yr[i] + bTrtYr*TrtYr[i]

}

# plot level model

for(j in 1:n){

logit(psi[j]) <- bMean[StandYr[j]]

Z[j] ~ dbin(psi[j], 1)

for(k in 1:3){

logit(p[j,k]) <- gInt

p.eff[j,k] <- Z[j]*p[j,k]

y[j,k] ~ dbin(p.eff[j,k], 1)

}

}

}

# ------------------------

# 2. helper functions

# ------------------------

# gen data

genData1 <- function(R, S, posttrt, det){

preTrtOcc <- 0.70

beta0 <- log(preTrtOcc/(1-preTrtOcc))

beta3 <- log((posttrt/(1-posttrt))/(preTrtOcc/(1-preTrtOcc)))

Dat <- expand.grid(Yr=c(0,1), Stand=1:R)

Dat$Trt <- ifelse(Dat$Stand <= R/2, 0, 1)

Dat$StandYr <- 1:nrow(Dat)

b0 <- rnorm(R, mean=0, sd=1)

Dat$b0 <- b0[Dat$Stand]

Dat$logitpsi <- with(Dat, beta0 + b0 + beta3*Trt*Yr)

Dat$psi <- 1/(1+exp(-Dat$logitpsi))

Dat2 <- data.frame(Yr = rep(Dat$Yr, each=S),

Stand = rep(Dat$Stand, each=S),

Trt = rep(Dat$Trt, each=S),

StandYr = rep(Dat$StandYr, each=S),

psi = rep(Dat$psi, each=S))

occ <- rbinom(nrow(Dat2), 1, Dat2$psi)

detection <- cbind(rbinom(nrow(Dat2), 1, det*occ),

rbinom(nrow(Dat2), 1, det*occ),

rbinom(nrow(Dat2), 1, det*occ))

zst <- apply(detection, 1, max)

test.data <- list(y=detection, R=R, N=R*2, n=R*S*2, Trt=Dat$Trt, Yr=Dat$Yr, TrtYr= Dat$Trt * Dat$Yr,

StandID=Dat$Stand, StandYr = Dat2$StandYr)

list(test.data=test.data, zst=zst, Z=occ, psi=Dat2$psi)

}

# fit model

fit1 <- function(dat, params, model, n.chains, n.thin, n.iter, n.burnin){

inits <- function(){list(Z=dat$zst)}

out <- jags(data=dat$test.data, inits=inits, parameters.to.save=params, model.file=model,

n.chains=n.chains, n.thin=n.thin, n.iter=n.iter, n.burnin=n.burnin)

out

}

# run sim

runSim <- function(sets, nsim, params, model, n.chains, n.thin, n.iter, n.burnin, dfile){

nsets <- nrow(sets)

for(i in 1:nsets){

R <- sets$R[i]

S <- sets$S[i]

posttrt <- sets$posttrt[i]

det <- sets$det[i]

for(j in 1:nsim){

dat.ij <- genData1(R=R, S=S, posttrt=posttrt, det=det)

fm.ij <- fit1(dat.ij, params=params, model=model, n.chains=n.chains, n.thin=n.thin, n.iter=n.iter, n.burnin=n.burnin)

fname <- paste("Nstand.", R, "_Nsub.", S, "_Posttrt.", posttrt, "_det.", det, "_sim.", j, ".csv",sep="")

write.csv(fm.ij$BUGSoutput$summary, paste(dfile, fname, sep=""))

}

}

}

# ------------------------------

# 3. set & run the conditions

# ------------------------------

sets <- expand.grid(R=c(20, 30, 40, 50, 60), S=c(5, 7, 9), posttrt=c(0.1, 0.3), det=c(0.15, 0.30, 0.50))

params <- c("aInt.mean", "bTrt", "bYr", "bTrtYr", "gInt")

nsim=500

system.time(temp <- runSim(sets=sets, nsim=nsim, params=params, model=model.h, n.chains=3, n.thin=10, n.iter=10000, n.burnin=5000, dfile="C:/"))
