# Supplementary material for: Evaluating Multi-Level Models to Test Occupancy State Responses of Plethodontid Salamanders
Source: PLoS One. 2015 Nov 30;10(11):e0142903. doi: 10.1371/journal.pone.0142903 (PMC4664280; doi:10.1371/journal.pone.0142903)
Supplement: S2 File — (DOCX) [file pone.0142903.s003.docx]

**S3 File:** R code for the simulation study for 'Multi-scale' BACI occupancy model.

#

# Simulation study for 'Multi-scale' BACI occupancy model

#

library(R2jags)

library(plyr)

# ------------------------------

# 1. JAGS model

# ------------------------------

# Multi-scale occupancy model

model.m <- function(){

alphaTrt ~ dnorm(0, 0.333)

alphaYr ~ dnorm(0, 0.333)

alphaTrtYr ~ dnorm(0, 0.1)

beta0 ~ dnorm(0, 0.333)

gamma0 ~ dnorm(0, 0.333)

a0.mean ~ dnorm(0, 0.1)

a0.var ~ dgamma(1, 1)

a0.tau <- 1/a0.var

for(i in 1:R){ # stands

a0[i] ~ dnorm(a0.mean, a0.tau)

}

for (i in 1:N){ # Stand-years

psi[i] <- a0[StandID1[i]] + alphaTrt*Trt[i] + alphaYr*Yr[i] + alphaTrtYr*TrtYr[i]

psip[i] <- 1/(1+exp(-psi[i]))

z[i] ~ dbern(psip[i])

}

for(j in 1:n){ # plots

theta[j] <- beta0

theta.eff[j] <- z[StandYr[j]]*(1/(1+exp(-theta[j])))

u[j] ~ dbern(theta.eff[j])

for(k in 1:V){ # visits

p[j,k] <- gamma0

p.eff[j,k] <- u[j] * (1/(1+exp(-p[j,k])))

y[j,k] ~ dbern(p.eff[j,k])

}

}

}

# ------------------------

# 2. helper functions

# ------------------------

# gen data

genData1 <- function(R, S, posttrt, detProb){

preTrtProb <- 0.95

subPlotProb <- 0.5

beta0 <- log(preTrtProb/(1-preTrtProb))

beta3 <- log((posttrt/(1-posttrt))/(preTrtProb/(1-preTrtProb)))

Dat1 <- expand.grid(Yr=c(0,1), Stand=1:R)

Dat1$Trt <- ifelse(Dat1$Stand <= R/2, 0, 1)

b0 <- rnorm(R, mean=0, sd=1)

Dat1$b0 <- b0[Dat1$Stand]

Dat1$logitpsi <- with(Dat1, beta0 + b0 + beta3*Trt*Yr)

Dat1$psi <- 1/(1+exp(-Dat1$logitpsi))

Dat1$StandOcc <- rbinom(nrow(Dat1), 1, Dat1$psi)

Dat2 <- data.frame(Yr=rep(Dat1$Yr, each=S),

Stand=rep(Dat1$Stand, each=S),

Trt=rep(Dat1$Trt, each=S),

StandOcc=rep(Dat1$StandOcc, each=S),

StandYr=rep(1:nrow(Dat1), each=S))

Dat2$SubPlotOcc <- rbinom(nrow(Dat2), 1, subPlotProb*Dat2$StandOcc)

zst <- (ddply(Dat2, c("Yr", "Stand"), function(x) data.frame(zst=max(x$SubPlotOcc))))$zst

detection <- cbind(rbinom(nrow(Dat2), 1, detProb*Dat2$SubPlotOcc),

rbinom(nrow(Dat2), 1, detProb*Dat2$SubPlotOcc),

rbinom(nrow(Dat2), 1, detProb*Dat2$SubPlotOcc))

ust <- apply(detection, 1, max)

test.data <- list(y=detection, R=R, N=R*2, n=R*S*2, V=3, Trt=Dat1$Trt, Yr=Dat1$Yr,

TrtYr=Dat1$Trt*Dat1$Yr, StandYr=Dat2$StandYr, StandID1=Dat1$Stand)

list(test.data=test.data, zst=zst, ust=ust, Z=Dat1$StandOcc, psi=Dat1$psi, U=Dat2$SubPlotOcc)

}

# fit model

fit1 <- function(dat, params, model, n.chains, n.thin, n.iter, n.burnin){

inits <- function(){list(z=rep(1, dat$test.data$N), u=rep(1, dat$test.data$n))}

out <- jags(data=dat$test.data, inits=inits, parameters.to.save=params, model.file=model,

n.chains=n.chains, n.thin=n.thin, n.iter=n.iter, n.burnin=n.burnin)

out

}

# run sim

runSim <- function(sets, nsim, params, model, n.chains, n.thin, n.iter, n.burnin, dfile){

nsets <- nrow(sets)

for(i in 1:nsets){

R <- sets$R[i]

S <- sets$S[i]

posttrt <- sets$posttrt[i]

detProb <- sets$detProb[i]

for(j in 1:nsim){

dat.ij <- genData1(R=R, S=S, posttrt=posttrt, detProb=detProb)

fm.ij <- fit1(dat.ij, params=params, model=model, n.chains=n.chains, n.thin=n.thin, n.iter=n.iter, n.burnin=n.burnin)

fname <- paste("Nstand.", R, "_Nsub.", S, "_Posttrt.", posttrt, "_det.", detProb, "_sim.", j, ".csv",sep="")

write.csv(fm.ij$BUGSoutput$summary, paste(dfile, fname, sep=""))

}

}

}

# ------------------------------

# 3. set & run the conditions

# ------------------------------

sets <- expand.grid(R=c(20, 30, 40, 50, 60), S=c(5, 7, 9), posttrt=c(0.3, 0.6), det=c(0.15, 0.30, 0.50))

params <- c("a0.mean", "alphaTrt", "alphaYr", "alphaTrtYr", "beta0", "gamma0")

nsim=500

system.time(temp <- runSim(sets=sets, nsim=nsim, params=params, model=model.m, n.chains=3, n.thin=10, n.iter=10000, n.burnin=5000, dfile="C:/"))
