# Supplementary material for: Evaluating Multi-Level Models to Test Occupancy State Responses of Plethodontid Salamanders
Source: PLoS One. 2015 Nov 30;10(11):e0142903. doi: 10.1371/journal.pone.0142903 (PMC4664280; doi:10.1371/journal.pone.0142903)
Supplement: S3 File — (DOCX) [file pone.0142903.s004.docx]

**S4 File:** R code for empirical analyses of Oregon slender and ensatina salamander occupancy, Oregon Cascades, US, 2013-2014.

#

# 'hierarchical' model used to fit empirical pre-treatment data

# for both OSS and ENES

#

model.1 <- function(){

aInt.mean ~ dnorm(0,0.333)

aInt.sd ~ dgamma(2, 1)

aInt.tau <- 1/(aInt.sd * aInt.sd)

aTFCL ~ dnorm(0, 0.25)

bDW ~ dnorm(0, 0.25)

bYear ~ dnorm(0, 0.25)

gInt ~ dnorm(0, 0.333)

gDate ~ dnorm(0, 0.25)

gDate2 ~ dnorm(0, 0.25)

gYear ~ dnorm(0, 0.25)

for(i in 1:R){

muStand[i] <- aInt.mean + aTFCL*TFCL[i]

bInt[i] ~ dnorm(muStand[i], aInt.tau)

}

for(j in 1:n){

logit(psi[j]) <- bInt[StandID[j]] + bDW*DW[j] + bYear*Year14[j]

z[j] ~ dbern(psi[j])

for(k in 1:3){

logit(p[j,k]) <- gInt + gDate*JD[j] + gDate2*JD[j]*JD[j] + gYear*Year14[j]

p.eff[j,k] <- z[j] * p[j,k]

y[j,k] ~ dbern(p.eff[j,k])

}

}

}
